# MERS-CoV RBD-mRNA Presents Better Immunogenicity and Protection than the Spike-mRNA

**DOI:** 10.3390/cells14231928

**Published:** 2025-12-04

**Authors:** Qian Liu, Abhishek K. Verma, Xiaoqing Guan, Shengnan Qian, Stanley Perlman, Lanying Du

**Affiliations:** 1 Institute for Biomedical Sciences, Georgia State University, Atlanta, GA 30303, USA; 2 Department of Microbiology and Immunology, University of Iowa, Iowa City, IA 52242, USA; 3 Department of Pediatrics, University of Iowa, Iowa City, IA 52242, USA

**Keywords:** coronavirus, MERS-CoV, spike, receptor-binding domain, neutralizing activity, protective efficacy

## Abstract

**Highlights:**

**What are the main findings?**

- Compared with MERS-CoV S-mRNA, MERS-CoV RBD-mRNA induced better antibody responses with broadly neutralizing antibodies against multiple MERS-CoV strains.
- MERS-CoV RBD-mRNA provided strong and durable protective efficacy against MERS-CoV challenge in a murine model.

**What are the implications of the main findings?**

- The RBD of MERS-CoV has great potential to serve as a critical target for the development of effective MERS-CoV vaccines.
- This study provides useful guidance for rational design of MERS-CoV vaccines.

**Abstract:**

Pathogenic Middle East respiratory syndrome CoV (MERS-CoV), first identified in Saudi Arabia in 2012, continues to pose a threat to public health. The trimeric spike (S) protein of MERS-CoV binds to the cellular receptor through the receptor-binding domain (RBD) in the S1 subunit to initiate virus entry and infection. Therefore, both the S protein and its RBD are targets for the development of MERS-CoV vaccines. Nevertheless, a direct comparison of the immune efficiency of S- and RBD-based MERS-CoV vaccines has not been made. Here, we compared two mRNA vaccines, respectively, targeting the S (S-mRNA) and RBD (RBD-mRNA) of MERS-CoV for their durable immunogenicity, neutralizing activity, and protective efficacy in a mouse model. Both mRNAs encapsulated with lipid nanoparticles (LNPs) maintained strong stability at various temperatures during the detection period. LNP-encapsulated RBD-mRNA elicited significantly higher and more durable antibodies than LNP-encapsulated S-mRNA, maintaining stronger and broadly neutralizing activity against the MERS-CoV original strain, as well as multiple variants containing key mutations within the RBD region. Importantly, RBD-mRNA provided durable protective efficacy against MERS-CoV infection in middle-aged mice, and this protection was associated positively with serum neutralizing antibody titers. Overall, this study identifies RBD-mRNA as an effective vaccine against MERS-CoV, with great potential for further development.

## 1. Introduction

The highly pathogenic Middle East respiratory syndrome coronavirus (MERS-CoV), first identified in Saudi Arabia in 2012, causes Middle East respiratory syndrome (MERS) disease [1]. As a zoonotic virus, MERS-CoV is transmitted to humans through an intermediate host, dromedary camels [2,3,4,5]. Unlike other highly pathogenic human CoVs such as severe acute respiratory syndrome CoV-2 (SARS-CoV-2) [6], MERS-CoV exhibits limited human-to-human transmissibility, meaning that it causes mainly sporadic infections in humans, with rare local outbreaks occurring in healthcare settings or community environments due to close contact [7,8,9]. Nine MERS cases were reported to the WHO between 1 March 2025 and 21 April 2025, of which two died. Seven were identified as a cluster in Riyadh, including six healthcare workers taking care of a MERS-CoV-infected patient [10]. As of 6 October 2025, about 2640 MERS cases, including 958 deaths, have been reported globally by health authorities, mostly in Saudi Arabia [11]. MERS-CoV continues to infect humans with a high mortality rate (>36%). Currently, there are no approved vaccines or therapeutic agents against MERS-CoV infection; therefore, continuous efforts are needed to develop countermeasures to prevent or treat MERS disease.

MERS-CoV has a single-strand RNA genome that encodes four structural proteins (spike (S), envelope, membrane, and nucleocapsid), among which the S protein plays a crucial role in viral infection and pathogenesis [12,13]. The native S protein is a trimer comprising S1 and S2 subunits [14]. MERS-CoV enters cells by binding to a cellular receptor, dipeptidyl peptidase-4 (DPP4), through the receptor-binding domain (RBD) in the S1 region; this is followed by the subsequent fusion of the virus and cell membrane through the S2 subunit [15,16,17,18,19]. Therefore, both the S protein and its RBD fragment serve as important targets for the development of effective vaccines and therapeutic antibodies [20,21]. Although a number of vaccines targeting the S and RBD fragment have been developed [22,23,24,25], a direct comparison of the immune efficiency of the S and the RBD-based MERS-CoV vaccines has not been made.

Here, we designed two mRNA vaccines to target, respectively, the S protein and RBD fragment of MERS-CoV. To maintain their trimeric conformation, we added a C-terminal trimeric motif foldon to each construct and encapsulated the synthesized mRNA within lipid nanoparticles (LNPs) for delivery into mice. The durable immunogenicity and neutralizing ability of these mRNA vaccines were compared for up to 5 months post-final immunization. Given that advanced age is a major risk factor for MERS-CoV infection, leading to increased disease severity and mortality in elder individuals [26,27], we evaluated the protective efficacy of both mRNA vaccines in elderly mice (middle-aged: >12 months), representing an adult population approaching old age.

## 2. Materials and Methods

### 2.1. Cells

HEK293T, Huh-7, and Vero E81 cells (ATCC, Manassas, VA, USA) were cultured in DMEM (Dulbecco’s Modified Eagle’s Medium, Thermo Fisher Scientific, Waltham, MA, USA) containing 1% penicillin/streptomycin (Thermo Fisher Scientific) and 10% Fetal Bovine Serum (FBS) (R&D Systems, Minneapolis, MN, USA) to support cell growth and prevent contamination. The cells were maintained at 37 °C in a 5% CO_2_ incubator, and observed daily for sterility, morphology, and adherence over a period of approximately 8–10 passages. HEK293F cells (Thermo Fisher Scientific) were cultured in serum-free medium (ESF-SFM) (Expression Systems, Davis, CA, USA) at 37 °C and 8% CO_2_ with constant shaking at 120× rpm rotation.

### 2.2. Design and Construct of mRNA Vaccines

S-mRNA and RBD-mRNA were designed and constructed as previously described [28,29]. Briefly, recombinant DNA sequences—encoding the RBD or S protein, respectively, of MERS-CoV—were amplified by PCR, using a codon-optimized S plasmid (containing two proline) of MERS-CoV (wildtype EMC2012 strain: GenBank accession number JX869059.2) as the template. A N-terminal signal peptide, tissue plasminogen activator (tPA), a C-terminal foldon trimeric sequence, and a C-terminal His_6_ tag were added during PCR amplification. The purified PCR products were ligated into an enzyme-digested pCAGGS vector using Seamless Cloning reagent (Vazyme; Nanjing, China). The recombinant plasmids also contain a 5′-terminal T7 promoter, a 5′-untranslated region (5’-UTR), and a 3′-UTR. The recombinant plasmids were sequenced using Psomagen DNA sequencing service to confirm correct insertions (Rockville, MD, USA).

### 2.3. Synthesis, Formulation and Characterization of mRNA Vaccines

The S-mRNA and RBD-mRNA of MERS-CoV were synthesized as previously described [28,29]. Briefly, the mRNAs were synthesized using the MEGAscript T7 Transcription kit (Thermo Fisher Scientific), in the presence of nucleosides (ATP, CTP, and GTP) and a modified nucleoside, pseudouridine-5′-O-triphosphate (Pseudo-UTP; APExBIO, Houston, TX, USA). In order to enhance translation efficiency and stability, these mRNAs were further capped at the N-terminus using the ScriptCap™ Cap 1 Capping System (CELLSCRIPT, Madison, WI, USA) and tailed at the C-terminus with a poly(A) tail using the A-Plus™ Poly(A) Polymerase Tailing Kit (CELLSCRIPT), following the manufacturer’s standard protocols. The Cap 1 Capping System contained ScriptCap™ 2′-O-Methyltransferase in order to form a Cap 1-RNA cap structure, ensuring approximately 100% capping efficacy at the 5′ end, whereas the A-Plus™ Poly(A) Polymerase Tailing Kit added a stable tail with about 150-length poly(A).

The synthesized S-mRNA and RBD-mRNA were encapsulated in LNPs using a previously described method [28,29]. Briefly, the synthesized mRNA in the PNI Formulation Buffer, or nuclease-free water as a control for LNPs, was formulated with a lipid mixture, GenVoy-ILM (Precision Nanosystems, Vancouver, BC, Canada) at a 3:1 ratio using the NanoAssemblr Benchtop Instrument (Precision Nanosystems). The LNP-formulated mRNA was further concentrated using Centrifugal Filters (10 kDa Amicon Ultra-15; EMDMillipore, Burlington, MA, USA), and stored at 4 °C until use. The particle size and PDI (Polydispersity Index) of the formulated mRNA-LNPs were measured using the DynaPro NanoStar II Light Scattering Detector (DLS) (WYATT Technology, Santa Barbara, CA, USA).

### 2.4. RNA Gel Electrophoresis

RNA gel electrophoresis was performed according to the protocol described below. Briefly, S-mRNA or RBD-mRNA was recovered from mRNA-LNP formulations using phenol–chloroform extraction. The recovered RNAs, as well as the pre-formulated RNA controls, were incubated with 2× RNA Loading Dye, and heated at 70 °C for 5 min. A 2% agarose gel was prepared in a buffer containing 50 mM of MOPS (pH 7.0), 1 mM of EDTA, and formaldehyde. Before loading RNA samples, the gel was pre-run in 1× MOPS–EDTA running buffer at 60 V for 30 min. Each RNA sample was then applied to the aforementioned gel and electrophoresed at 60 V for 1 h. After separation, the gel was stained with ethidium bromide, and then de-stained in water for 10 min.

### 2.5. Construct, Expression and Purification of Recombinant Proteins

Recombinant DNAs encoding the RBD or S protein of MERS-CoV were amplified by PCR, and the template was the plasmid encoding the codon-optimized S sequence of MERS-CoV (wildtype EMC2012 strain), mentioned above. The purified PCR products, which contained a C-terminal foldon sequence and a C-terminal His_6_ tag, were ligated into an enzyme-digested plenti vector using the Seamless Cloning reagent (Vazyme). The sequence-confirmed recombinant plasmids were transiently transfected into HEK293F cells using a transfection reagent, Polyethylenimine Hydrochloride (PEI) (Polysciences, Warrington, PA, USA), and the mass ratio of PEI–plasmid was 3:1. Five days later, the culture supernatants were centrifuged at 6000× *g* for 20 min, and the related recombinant proteins were purified using Ni-NTA Superflow (Qiagen, Germantown, MD, USA). The purified proteins were subsequently concentrated with Centrifugal Filters (10 kDa MWCO Amicon Ultra-15; EMDMillipore) using PBS (pH 7.4) buffer, to which we added 137 mM of NaCl, 2.7 mM of KCl, 8 mM of Na_2_HPO_4_, and 1.47 mM of KH_2_PO_4_. These purified proteins were confirmed for accuracy, precision, and repeatability, which were used for the ELISA method described below.

### 2.6. Animal Vaccination and Sample Collection

Female C57BL/6 (B6) mice at the age of 20–25 weeks old were used for immunization in the study. The mice were housed in well-ventilated cages measuring 34.3 cm × 29.2 cm × 15.5 cm (length × width × height). Each cage was filled with 1/8-inch Bed-o’Cob bedding. The mice were provided with commercial rodent food and water. They were kept on a 12 h dark cycle and had access to enrichment devices. Pathogen-free B6 mice (5 mice/group) were randomly assigned to three groups, and immunized intradermally (i.d.) with LNP-formulated S-mRNA, RBD-mRNA (10 µg/100 µL/mouse), or the LNP control (100 µL/mouse). The immunized mice were boosted twice with the same immunogen at 3-week intervals. Blood samples were collected through facial bleeding 10 days, or 1, 3, and 5 months, after the last immunization as well as before virus challenge (i.e., about 10 months after the last immunization). Specifically, peripheral blood was collected via facial (submandibular) vein puncture, as this method allowed for the frequent collection of small volumes of blood over extended periods, with faster sampling and minimal adverse clinical signs. On average, approximately 0.2 mL of blood was obtained per sample by targeting the vascular bundle located at the back of the jaw of mouse [30]. The collected serum samples were further analyzed for MERS-CoV-specific antibodies and/or neutralizing antibodies using ELISA and the pseudovirus neutralization assay.

### 2.7. ELISA

IgG and its subtype antibodies (IgG1 and IgG2c), specific to the RBD or S protein of MERS-CoV, were evaluated by ELISA in mouse sera collected above. Briefly, ELISA plates were coated with the abovementioned purified MERS-CoV RBD or MERS-CoV S protein (1 μg/mL) in a coating buffer (0.1 M NaHCO_3_, pH 8.2), and incubated at 4 °C overnight. The plates were blocked with 1% bovine serum albumin in PBST buffer (PBS supplied with 0.1% Tween-20) at 37 °C for 1 h, and washed with PBST buffer at least three times. The plates were subsequently incubated with serially diluted mouse sera at 37 °C for 2 h, and washed again with PBST buffer at least three times. The plates were further incubated with secondary antibodies conjugated with horseradish peroxidase (HRP), including anti-mouse IgG (1:10,000), anti-mouse IgG1 (1:8000), and anti-mouse IgG2c (1:8000) (Thermo Fisher Scientific), respectively, at 37 °C for 1 h. Afterwards, the plates were washed with PBST buffer for six times, and then treated with 3,3′,5,5′-Tetramethylbenzidine (TMB) substrate (MilliporeSigma, Burlington, MA, USA) at room temperature for 3 min, after which the reaction was terminated by H_2_SO_4_ (1 N). The optical density at 450 nm (OD_450_) was measured by Microplate Reader (Cytation 7, BioTek Instruments, Winooski, VT, USA). The OD_450_ values were examined using nonlinear regression (dose–response inhibition: log (inhibitor) vs. response (three parameter)), and the antibody titer was determined using a cutoff value at four times of the value of the no-serum blank.

### 2.8. MERS Pseudovirus Preparation and Neutralization Assay

MERS-CoV pseudoviruses were prepared as previously described [28]. Briefly, pseudoviruses encoding the S protein of the MERS-CoV original strain, and variants of the S protein containing L506F, L507P, A520S, E536K, and D537E mutations, respectively, in the RBD region, were prepared by the co-transfection of HEK293T cells with each of the aforementioned recombinant S plasmids of MERS-CoV and an HIV-Luc plasmid using the PEI transfection reagent mentioned above. At 6–8 h post-transfection, the medium was changed to a fresh cell culture medium supplied with 10% FBS. At 72 h post-transfection, the cell culture supernatants, which contained generated pseudovirus, were collected by centrifugation at 2000× *g* rpm for 10 min to remove cell debris.

A pseudovirus neutralization assay was then performed to measure serum neutralizing antibodies, as previously described [28,31]. Briefly, each pseudovirus generated above was incubated with serially diluted mouse sera at 37 °C for 1 h. The serum–virus mixture was added to Huh-7 cells, which were pre-seeded in 96-well culture plates. At 24 h later, fresh medium was added to the cells, followed by the continual culturing of the cells for 48 h. The cells were then lysed with a cell lysis buffer (Promega, Madison, MI, USA), and the cell lysates were transferred to white microplates. Luciferase substrate (Promega) was added to the plates, and relative luciferase activity was measured using the Microplate Reader (Cytation 7, BioTek Instruments). The pseudovirus neutralizing antibody titer was reported as a 50% neutralization titer (NT_50_).

### 2.9. Virus Challenge and Protection Evaluation

MERS-CoV challenge studies, as well as subsequent sample collection and evaluation, were performed as previously described with some modification [28,31]. Briefly, ten months post-last immunization, mice were intranasally (i.n.) transduced with Adenovirus 5 (Ad5)-hDPP4 (Ad5CMV/hDPP4-myc-flag vector; 2.5 × 10^8^ focus-forming unit). Five days after transduction, these mice were i.n. challenged with MERS-CoV (EMC2012 strain, 10^5^ plaque-forming unit (PFU)/50 μL/mouse). Ad5-hDPP4-transduced, MERS-CoV-infected wildtype B6 mice are not expected to show mortality and severe pathological changes, and hDPP4 expression is mainly restricted to the lungs [32]; as such, only lung viral titers were detected after infection. Lungs were collected 3 days after challenge, and viral titers were measured by a plaque assay, as described below. Briefly, lung tissues were homogenized, and serially diluted supernatants were incubated with Vero E81 cells (containing DMEM medium) at 37 °C for 1 h. The medium was removed, and the cells were covered with agarose (0.6%), followed by continuous culture at the same conditions for three days. The overlays were removed from the cells, and the plaques in each well were shown by staining with crystal violet (0.1%) (Fisher Scientific, Hampton, NH, USA). Lung viral titers were determined as the PFU/mL of lung tissues.

### 2.10. Statistical Analysis

GraphPad Prism 9 statistical software was used to analyze experimental data. Ordinary one-way ANOVA (Tukey’s multiple comparison test) was used to assess the statistical significance of the respective antibody titers, neutralizing antibody titers, and viral titers among different groups. Asterisks are used to indicate significance levels: *, **, ***, and **** illustrate *p* values of less than 0.05, 0.01, 0.001, and 0.0001, respectively.

## 3. Results

### 3.1. Design and Characterization of mRNA Vaccines

**Figure 1:**
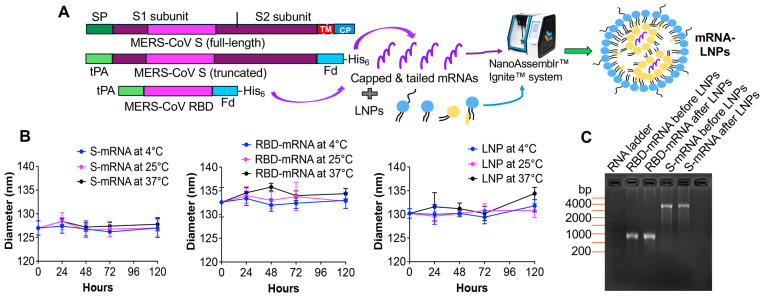
The design and characterization of MERS-CoV S-mRNA and RBD-mRNA vaccines. (**A**) The design, synthesis, and formulation of mRNAs. The full-length S protein of MERS-CoV contains the S1 and S2 subunits with its original signal peptide (SP) on the N-terminus, whereas the S2 subunit includes the transmembrane domain (TM) and cytoplasmic tail (CP), among other components. Each mRNA encodes the tPA signal peptide and a truncated S protein (i.e., excluding TM and CP regions), or its RBD fragment of MERS-CoV, with a C-terminal foldon (Fd) trimeric motif and a His_6_ tag. The synthesized mRNAs, capped at the N-terminus and tailed (with a Poly(A) sequence) at C-terminus, were encapsulated with lipid nanoparticles (LNPs) using the NanoAssemblr Ignite nanoparticle formulation system instrument to form mRNA-LNPs. (**B**) The determination of the stability of LNP-encapsulated S-mRNA or RBD-mRNA by measurement of the particle sizes using DynaPro NanoStar II Light Scattering Detector (DLS, WYATT Technology). Each mRNA was, respectively, stored at 4 °C, 25 °C, and 37 °C for 24, 48, 72, and 120 h, followed by the measurement of the particle sizes (hydrodynamic diameter: nm). LNP (without encapsulation with mRNA) was included as a control. The data in (**B**) indicates the mean ± standard deviation of the mean (s.e.m) of triplicate wells. (**C**) The agarose gel electrophoresis of RNAs extracted from the LNP-encapsulated mRNA samples (after LNPs). RNA samples without formulation with LNPs (before LNPs) were added as controls. Each RNA sample was loaded with 1 μg per well. The RNA ladder is shown on the left. The experiments were repeated once, with similar results.

We designed two mRNA vaccines (RBD-mRNA and S-mRNA), encoding the RBD fragment and truncated S protein, respectively, of MERS-CoV (Figure 1A). Each mRNA was synthesized as described in the Materials and Method section, being capped at the N-terminus and tailed with poly(A) at the C-terminus, followed by encapsulation with LNPs to increase mRNA’s stability and translation efficiency (Figure 1A). The vaccines were then tested to assess stability and immunogenicity as described below. As expected, the LNP-encapsulated S-mRNA and RBD-mRNAs were about 126–128 nm and 132–136 nm in size, respectively; this size was maintained at 4 °C, 25 °C, and 37 °C for up to 120 h during the detection period (Figure 1B). The PDI for RBD-mRNA-LNPs was less than 0.0005, and for S-mRNA-LNPs and control LNPs, it was less than 0.0001. The formulated mRNAs displayed high integrity, as validated by the RNA gel electrophoresis after being extracted from the mRNA-LNPs (Figure 1C). These results demonstrate that the synthesized, LNP-formulated mRNA vaccines show high integrity and strong stability under different temperatures.

### 3.2. MERS-CoV RBD-mRNA Elicited Better Antibody Responses than MERS-CoV S-mRNA

**Figure 2:**
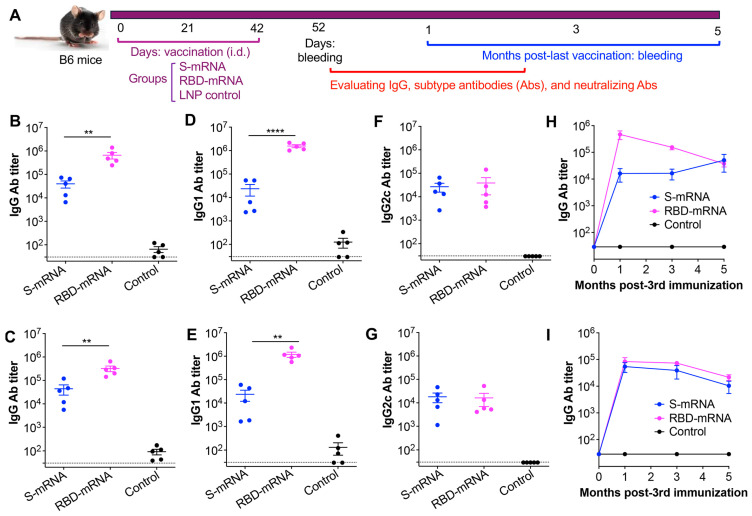
The measurement of MERS-CoV S or RBD-specific antibody responses induced by the MERS-CoV mRNA vaccines. (**A**) Immunization schedules and antibody testing. B6 mice were injected with S-mRNA, RBD-mRNA, or LNP control 3 times at 3-week intervals. Sera were collected 10 days post-last dose or once every other month for up to 5 months to test for total IgG antibody (Ab) response, IgG subtype Abs (IgG1 and IgG2c), or neutralizing Abs. The measurement of IgG antibody (**B**,**C**) and its subtypes (IgG1 and IgG2c) (**D**–**G**) specific to MERS-CoV RBD (**B**,**D**,**F**) or S (**C**,**E**,**G**) protein by ELISA. Sera collected 10 days post-last dose were used for this testing. The dashed lines indicate the detection limit. The evaluation of durable IgG Abs specific to MERS-CoV RBD (**H**) or S (**I**) protein by ELISA. Sera collected from 1, 3, and 5 months post-last dose were used for this testing. The ELISA plates were coated with RBD or S protein of MERS-CoV, and the Ab titer was presented as the mean ± s.e.m. of five mice in each group. Ordinary one-way ANOVA (Tukey’s multiple comparison test) was used to compare statistical significance among different groups. ** and **** indicate *p* < 0.01 and *p* < 0.0001, respectively. The experiments were repeated once, with similar results.

To evaluate the immunogenicity of the above-designed vaccines, B6 mice (20–25-week-old) were immunized with LNP-encapsulated RBD-mRNA, S-mRNA, or an LNP control, and boosted twice at 3-week intervals. Serum was collected 10 days after the last immunization (Figure 2A), and MERS-CoV RBD- or S-specific IgG antibodies (and their IgG1 and IgG2c subtypes) were measured by ELISA. Notably, RBD-mRNA induced a significantly higher titer of IgG antibodies or IgG1-subtype antibodies than S-mRNA; these antibodies were specific for either the RBD or the S protein of MERS-CoV (Figure 2B–E). Both RBD-mRNA and S-mRNA vaccines elicited a similar level of IgG2c subtype antibodies specific for the RBD and S proteins of MERS-CoV (Figure 2F,G). To further evaluate the durable immunogenicity of these vaccines, serum samples were collected every other month (for up to 5 months) after the last immunization, and MERS-CoV S- or RBD-specific IgG antibody responses were measured in an ELISA. Overall, RBD-mRNA induced stronger antibody responses than S-mRNA specific for the RBD of MERS-CoV, particularly at 1 and 3 months post-final immunization; of note, both RBD-mRNA and S-mRNA elicited a similar level of IgG antibodies specific for the S protein of MERS-CoV at 1, 3, and 5 months post-final immunization (Figure 2H,I). By contrast, the LNP control elicited only a background level of MERS-CoV S- or RBD-specific antibody responses (Figure 2B–I). These results identify RBD-mRNA and S-mRNA as effective immunogens that induce durable MERS-CoV S- and RBD-specific antibody responses, with RBD-mRNA eliciting better IgG and subtype antibodies than S-mRNA.

### 3.3. MERS-CoV RBD-mRNA Induced Stronger and Broader Neutralizing Antibodies than MERS-CoV S-mRNA

**Figure 3:**
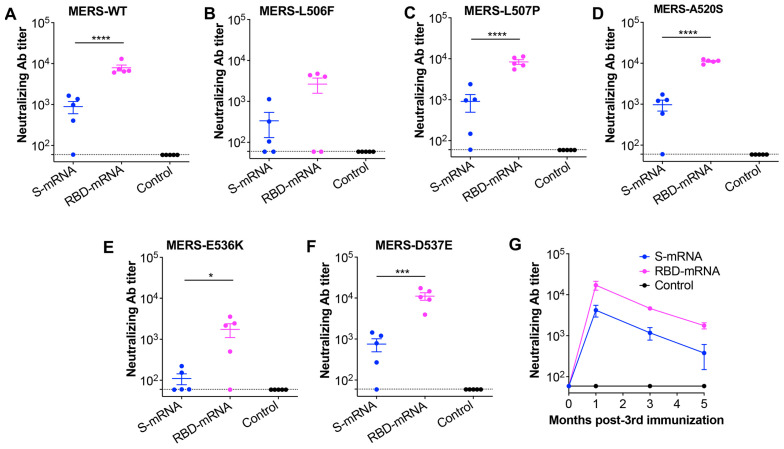
The evaluation of neutralizing antibodies induced by the MERS-CoV mRNA vaccines. Mouse sera collected 10 days post-last immunization of S-mRNA, RBD-mRNA, or LNP control were tested by the pseudovirus neutralization assay for broadly neutralizing antibodies (Abs) against the pseudotyped MERS-CoV original strain (MERS-WT) (**A**) or its variant strains, including MERS-L506F (**B**), MERS-L507P (**C**), MERS-A520S (**D**), MERS-E536K (**E**), and MERS-D537E (**F**), which contain key mutations in the RBD region. The dashed lines indicate the detection limit. (**G**) The evaluation of durable neutralizing Abs against pseudotyped MERS-WT strain by pseudovirus neutralization assay. Sera collected from 1, 3, and 5 months post-last immunization were used for this testing. The 50% neutralizing Ab titer (NT_50_) was determined based on the serum dilution at which 50% of the pseudotyped virus was neutralized. The neutralizing Ab titer is presented as the mean ± s.e.m. of five mice in each group. Ordinary one-way ANOVA (Tukey’s multiple comparison test) was used to compare statistical significance among different groups. *, *** and **** indicate *p* < 0.05, *p* < 0.001 and *p* < 0.0001, respectively. The experiments were repeated once, with similar results.

To compare the ability of the RBD-mRNA and S-mRNA vaccines to elicit effective neutralizing antibodies against MERS-CoV, sera collected at 10 days post-final immunization were tested for neutralizing activity against the pseudotyped MERS-CoV original strain (WT) and its variants (Figure 2A). Notably, RBD-mRNA induced significantly higher titers of neutralizing antibodies against five out of the six MERS-CoV strains tested, including the WT strain and variants carrying the L507P, A520S, E536K, and D537E mutations in the RBD region (Figure 3A–F). To further evaluate the durability of neutralizing antibodies against MERS-CoV infection, sera collected every other month post-final immunization were tested against the pseudotyped original WT strain (Figure 2A). Overall, RBD-mRNA induced higher neutralizing antibody titers than the S-mRNA at 1, 3, and 5 months post-final immunization (Figure 3G). These results suggest that RBD-mRNA from MERS-CoV elicits stronger and more durable neutralizing antibody responses than S-mRNA against multiple strains of MERS-CoV.

### 3.4. RBD-mRNA Provided Durable Protective Efficacy Against MERS-CoV in Middle-Aged Mice and the Protection Was Positively Associated with Serum Neutralizing Antibodies

**Figure 4:**
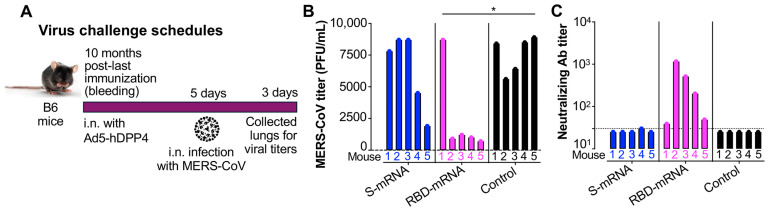
The evaluation of protective efficacy of the MERS-CoV mRNA vaccines. (**A**) Ten months post-last immunization of S-mRNA, RBD-mRNA, or LNP control, mice were intranasally (i.n.) transduced with Adenovirus 5 (Ad5)-hDPP4 vector, and five days later, they were i.n. challenged with MERS-CoV original strain (EMC2012, 10^5^ plaque-forming unit (PFU)/50 μL/mouse), followed by the testing of viral titers in the lungs 3 days later. (**B**) The evaluation of viral titers (PFU/mL) in the lungs of challenged mice by plaque assay. (**C**) The evaluation of serum neutralizing antibodies of mice before challenge. Sera collected before challenge were tested for neutralizing antibody (Ab) by pseudovirus neutralization assay against the pseudotyped MERS-CoV original strain (MERS-WT). The 50% neutralizing Ab titer (NT_50_) was determined based on the serum dilution at which 50% of the pseudotyped virus was neutralized. The data in (**B**,**C**) indicates the individual values of five mice in each group (mouse No. 1–5). The dashed line in (**C**) indicates the detection limit. Ordinary one-way ANOVA (Tukey’s multiple comparison test) was used to compare statistical significance among different groups. * indicates *p* < 0.05. The experiments were repeated once, with similar results.

The durable protective efficacy of RBD-mRNA and S-mRNA vaccines against MERS-CoV infection was evaluated in immunized B6 mice about 10 months after the final immunization. Mice were transduced with the Ad5-hDPP4 vector and then challenged with MERS-CoV 5 days later, followed by the measurement of viral titers in the lungs after further 3 days (Figure 4A). RBD-mRNA protected mice from MERS-CoV challenge, resulting in significantly lower viral titers than those measured in mice receiving the LNP control (Figure 4B). Next, we assessed the association between protective efficacy induced by the mRNA vaccines and the corresponding neutralizing antibody titers. Overall, the higher the neutralizing antibody titer in the RBD-mRNA-vaccinated mouse serum, the lower the viral titer in the lungs after challenge (Figure 4B,C). By contrast, the background level of neutralizing antibodies in control mice injected with LNPs resulted in high viral titers in the lungs post-challenge (Figure 4B,C). These results demonstrate the durable protective efficacy induced by the RBD-mRNA vaccine in middle-aged mice (>12 months) and show that this protection was positively associated with serum neutralizing antibody titers.

## 4. Discussion

mRNA vaccine technology is an innovative approach to the rapid development of effective vaccines against emerging and reemerging viral diseases, resulting in the fast approval of at least two mRNA vaccines against SARS-CoV-2 infection during the initial stages of the COVID-19 pandemic [33,34,35,36]. The advantages of mRNA vaccines over other vaccine types include rapid synthesis (within several days) and quick delivery (without integrating into cellular genomes), high efficiency, strong adaptability, good overall safety profile, and scalable manufacturing capability [37,38,39]. Despite the above strengths, mRNA technology has some weaknesses that need to be addressed appropriately [38,40]. For example, naked mRNAs are generally unstable and are easily degraded. In addition, mRNAs may cause potential side effects due to the increased secretion of proinflammatory cytokines or chemokines associated with innate immune activation. To increase the stability and translation efficiency of mRNAs, and eliminate other potential side effects, modified nucleosides (such as Pseudo-UTP, s2U, m6A, m5C, m5U, or m7G) are added during synthesis; the synthesized mRNAs are then encapsulated within LNPs for delivery [41,42]. The metabolism of mRNAs, such as re-adenylation by the TENT5A poly(A) polymerase, may stabilize target mRNAs, enhancing mRNA expression in cells and improving the in vivo efficacy of mRNA vaccines [43].

A few mRNA vaccines have been developed to target the S protein of MERS-CoV. For example, a triplex mRNA vaccine encoding S proteins of MERS-CoV, the SARS-CoV-2 Delta variant, and SARS-CoV elicited MERS-CoV S-specific antibodies and moderate anti-MERS-CoV neutralizing antibody titers in mice in a dose-dependent manner, with the antibody titer against MERS-CoV being lower than that against SARS-CoV-2 and SARS-CoV S proteins [44]. By contrast, duplex mRNAs encoding the MERS-CoV S protein and the SARS-CoV-2 Delta S protein or SARS-CoV S protein exhibited stronger anti-MERS-CoV neutralizing activity [44]. Sequential and cocktail immunizations of mice with LNP-mRNAs encoding the S proteins of MERS-CoV, SARS-CoV-2 (Omicron or Delta variant), and SARS-CoV induced immune responses against all CoVs tested, with the cocktail immunization eliciting higher neutralizing antibody titers and stronger cellular immune responses [45]. The encapsulation of MERS-CoV S-targeting mRNA within nanoporous aluminosilicate instead of LNPs also induced S-specific antibody responses in mice, but the antibody titer was lower than that induced by S-DNA with or without nanoporous aluminosilicate [46]. Other types of vaccine, such as DNA, viral vector, subunit, nanoparticle, or virus-like particle vaccines, which target the S protein of MERS-CoV S protein, have been studied [23,24,47,48,49,50,51,52], with the S-based DNA and viral vectored vaccines being evaluated in clinical trials. However, none of these vaccines have been approved for use in humans [53,54,55,56].

Very few mRNA vaccines have been reported that target the RBD fragment of the MERS-CoV S protein. For instance, a mRNA vaccine was designed to contain putative immunogenic epitopes, such as four cytotoxic T lymphocyte epitopes, four helper T lymphocyte epitopes, and six linear B cell epitopes, of the RBD regions of MERS-CoV, SARS-CoV-2, and SARS-CoV; this structure was predicted by computational design and analysis of its binding affinity by molecular docking [57]. In silico immune simulation analysis indicated that this mRNA vaccine may potentially induce specific antibody responses and cellular immune responses against the test CoVs, but this would need to be confirmed in animal models. Previously, we designed an mRNA targeting the RBD of MERS-CoV (without a C-terminal trimeric motif), and demonstrated its ability to induce neutralizing antibodies and cellular immune responses that protected mice from challenge with MERS-CoV [28]. In addition to mRNA vaccines, a variety of other types of vaccines targeting the RBD of MERS-CoV have been reported, including subunit, viral vector, nanoparticle, bacterium-like particle, and virus-like particle vaccines [22,31,58,59,60,61].

Although mRNA vaccines targeting the RBD and S proteins of MERS-CoV have been tested separately, it is unclear which is superior and can be considered an ideal target for the development of effective MERS-CoV vaccines. To fill in this gap, we designed two mRNA vaccines, targeting the S protein and RBD fragment of MERS-CoV, respectively.

A C-terminal foldon motif was added to each mRNA construct with the goal of forming a trimeric conformational structure. The mRNAs were synthesized in the presence of modified nucleoside Pseudo-UTP; the aim was to increase their stability and translation efficiency. Studies revealed a potential relationship between the size of LNPs and the immunogenicity of mRNA-LNPs in mice, indicating that LNPs with small diameters (around 64 nm) led to less immunogenic LNP-formulated mRNA vaccines [62]. Our results indicated that the LNPs formulated with either S-mRNA or RBD-mRNA were within the size range (around 81–146 nm) of nanoparticles, which showed a relatively better delivery and improved immune response in immunized mice [62].

The i.d. route was selected for mouse immunization since it was an optimal route in our previous study for inducing the highest immune responses and the production of protective neutralizing antibodies [28]. Generally, the skin is a highly immunoreactive tissue rich in antigen-presenting cells, which play key roles in initiating strong adaptive immune responses. Thus, delivering mRNA vaccines via an i.d. route would potentially enable a robust immune response with a lower dose of mRNA. Also, the i.d. route provides better accessibility for local monitoring and dose sparing, potentially reducing systemic reactogenicity [63]. Of note, the vaccine did not cause any damage at the injection site. An initial intradermal lump was observed following injection, which disappeared within one day. No redness, rash, or lesions were observed or detected at the injection sites, indicating that no pathological changes occurred solely by vaccination.

A variety of cell-based in vitro assays have been applied to CoV studies [64]. These assays employ either pseudovirus systems, relying on a luciferase reporter in target cells expressing the specific viral receptor, or live-virus methods, such as plaque assays and cytopathic effect assays, for the evaluation of the neutralizing capacity of vaccine-induced antibodies or therapeutic agents, and/or the measurement of viral titers for samples from virus-challenged animals [31,50,65,66,67]. Other strategies include cell–cell fusion assays targeting viral surface protein, particularly the S2 subunit, by utilizing two cell lines expressing the S protein and the corresponding receptor, respectively [68]. T-cell functional assays, cell-based ELISA, and ELISpot assays are often applied to evaluate cellular immunity following vaccination or virus infection. Additional cell-based approaches, such as protease inhibition and RNA-dependent RNA polymerase inhibition assays, air–liquid interface cultures of human airway epithelial cells used for studying CoV infection and immune interaction, lung organoids, and organ-on-a-chip platforms [64,69,70,71,72,73], are not suitable for evaluating vaccine-induced immune responses and protection; this is particularly true for those based on the viral S protein or its fragments. Here, by the use of cell-based pseudovirus and live virus-based plaque assays and other in vitro assays, such as ELISA, the durable immunogenicity, neutralizing activity, and protective efficacy of the well-characterized, LNP-encapsulated S-mRNA and RBD-mRNA vaccines were compared in samples from immunized mice. We found that, compared with trimeric S-mRNA, trimeric RBD-mRNA elicited better and more durable IgG and/or IgG1-subtype antibody responses that broadly neutralized multiple strains of MERS-CoV infection. In addition, the RBD-mRNA provided middle-aged mice with significant protection against MERS-CoV challenge, and the level of protection was positively associated with neutralizing antibody titers. Although no direct comparison was performed using RBD-mRNA with or without a trimeric structure, it appears that the trimeric RBD-mRNA elicited better, or at least similar, immune responses than the non-trimeric RBD-mRNA and provided similar protection against MERS-CoV infection [28].

The present study did not investigate the ability of the produced mRNA vaccines, including the trimeric RBD-mRNA, in eliciting specific cellular immune responses. Our previous study indicated that the non-trimeric MERS-CoV RBD-mRNA indeed elicited effective T follicular helper cell, germinal center B-cell, and RBD-specific B-cell and CD4^+^/CD8^+^ T-cell responses in the vaccinated mice [28,74]. It is therefore expected that the trimeric RBD-mRNA sharing the same RBD sequence as the non-trimeric RBD-mRNA would induce similarly efficient cellular immune responses specific to the RBD of MERS-CoV. Of note, MERS-CoV RBD-targeting antibodies mainly block the RBD from binding its cellular receptor DPP4, as reported in the cell and non-cell-based in vitro assays, such as the flow cytometry assay, ELISA, and the binding competition assay [75,76]. In addition to directly blocking S1/RBD-mediated receptor binding, antibodies included by MERS-CoV S-mRNA could also possibly inhibit virus infection through other cellular mechanisms, such as blocking S2-mediated cell–cell fusion. This warrants further investigation. Future studies could also be conducted to evaluate and compare the ability of the designed mRNA vaccines in inducing MERS-CoV-specific cellular responses and other functions in target cells and immunized mice using a number of the cell-based assays described above. Additional studies could be conducted to determine the role of cellular immune responses, in addition to the function of antibody responses, in protective/cross-protective efficacy against variant MERS-CoV infections. Further studies should evaluate the immunogenicity and/or protective efficacy of the vaccines in other small and large animal models to confirm their efficiency.

## 5. Conclusions

Overall, this study shows that RBD-mRNA elicited greater antibody responses, particularly neutralizing antibodies, and stronger protective efficacy against MERS-CoV than S-mRNA, indicating that the RBD could serve as an ideal target for the development of effective MERS vaccines.

## Data Availability

All related data is presented in this study. Relevant materials or supplies will be made available under a material transfer agreement. No code was used in this study.

## Abbreviations

The following abbreviations are used in this manuscript:

CoV	Coronavirus
DPP4	Dipeptidyl peptidase-4
ELISA	Enzyme-linked immunosorbent assay
i.d.	Intradermal
i.n.	Intranasal
LNPs	Lipid nanoparticles
MERS-CoV	Middle East respiratory syndrome CoV
RBD	Receptor-binding domain
S	Spike
SARS-CoV-2	Severe acute respiratory syndrome CoV-2
